# Gonadal cycle, reproductive indices and detection of parasitism in the clam *Ameghinomya antiqua* in natural beds of importance for fisheries

**DOI:** 10.1371/journal.pone.0266538

**Published:** 2022-04-08

**Authors:** Roberto Jaramillo, Valentina Prida, Pedro S. Rubilar, Leyla Cardenas, Valeria Prieto, Marcela P. Astorga

**Affiliations:** 1 Instituto de Ciencias Marinas y Limnológicas, Universidad Austral de Chile, Isla Teja, Valdivia, Chile; 2 Instituto de Acuicultura, Universidad Austral de Chile, Sede Puerto Montt, Casilla, Puerto Montt, Chile; 3 Magister en Ecología Aplicada, Universidad Austral de Chile, Isla Teja, Valdivia, Chile; 4 Instituto de Ciencias Ambientales y Evolutivas, Facultad de Ciencias, Universidad Austral de Chile, Isla Teja, Valdivia, Chile; 5 Centro FONDAP de Investigación en Dinámica de Ecosistemas Marinos de Altas Latitudes (IDEAL), Valdivia, Chile; University of California Riverside, UNITED STATES

## Abstract

The clam *Ameghynomia antiqua* is a highly important resource for fisheries due to its high catches volume. It is the bivalve mollusc with the highest fisheries landings from natural beds on the Pacific coast of southern South America; however, studies of the reproductive conditions of this species are scarce and date back many years. The object of the present work was to evaluate the reproductive characteristics of the species, analysing its gametogenic and gonadal cycle, and reproductive indices, in fishery locations that present the natural beds with the highest fisheries catches, as well as parasite loads in the species. The gonads of the individuals were sampled monthly over a year and classified into one of three states called: “in development”, “ripe” and “spawned”. Synchrony between the sexes was observed in the indicators of the Gonadosomatic Index and Condition Index in each of the locations, although no synchrony was observed between locations. In the gametogenic cycle, the "ripe" state was observed in females in spring-summer, followed by rapid recovery to new development of the gonads; in males the "ripe" state was observed throughout the year. It was observed that males entered the “spawned” state one month ahead of females. The presence of digenean parasites in the state of metacercariae was detected in the gonads and mantle. No significant differences were found in the prevalence or intensity of infection when analysed by sex and month. The metacercariae were identified, by sequencing of three DNA regions, as belonging to the clade shared by species of the genus *Parvatrema* and close to the *Gymnophalloides*; both these genera belong to the family Gymnophallidae of the superclass Digenea. Infection was observed to reduce the gonadal tissue, in some cases causing castration. This is the first record of the presence of these parasites of *A*. *antiqua*, with genetic identification at genus level. These results are relevant for act proper management of this resource, which is important for fishing.

## Introduction

Study of the gonadal cycle of any species provides necessary information on population reproductive period, to allow predictions about recruitment, the feasibility of obtaining seeds, and closed seasons [1, 2]. The gonadal maturation and reproductive cycle of bivalve molluscs is related to endogenous factors of the species, such as stored nutrients, genetic heritage, and hormonal factors [3–8]. Nevertheless, exogenous factors exert great influence in regulating the gametogenic cycle of marine bivalves; such as temperature, and food availability and quality [9–14], but light, tides and salinity may also play a role [15]. Previous reports on factors affecting reproduction in bivalves suggest longer spawning periods in lower latitude populations, where the constant supply of phytoplankton facilitates larval survival during most of the year [16]; in high latitude populations the phytoplankton supply is seasonal [17], restricting larval survival to a shorter period.

Clam production and aquaculture are important economic resources in a worldwide context. Clams represent 33% of the global production of the marine bivalve fisheries [18]. Specifically, the clam *Ameghinomya antiqua* (King, 1832) is distributed along both the Pacific and Atlantic coasts of the southern cone of South America. On the Pacific coast this species is of great significance in the benthic communities of the Chilean littoral zone [19] due to its economic importance, since the largest catches by value are landed by artisanal fishermen in southern Chile. In 2019, the fisheries landings were 12,000 tons [20]; however, a decade earlier, fisheries landings of 20,000 tons were reported. It is therefore important to obtain more information about this resource to ensure proper population management and maintenance over time. There are currently no laws governing extraction of this species; this absence of legislation may be responsible for the steadily increasing extraction pressure to which it has been subjected in recent years. This has generated a reduction in the size of its natural banks and consequently a reduction in its fishing due to the lower availability of the resource. Basic knowledge of its reproductive biology, such as defining its gametogenic cycle and reproductive indices, is crucial for proper management of this fishery. In the area of reproductive biology, very few studies have been published about its gonadal cycle [19, 21, 22]; which are from more than 25 years ago and with different results: for example, one study on the southern Pacific coast reported a continuous gonadal cycle with at least three main spawning periods [19], while another reported a seasonal cycle with spawning between November and February [21]. Asynchrony in the spawning period has been observed in other species of bivalves on the Pacific coast of South America, such as *Perumytilus purpuratus* [23, 24], with one of these studies reporting a semi-annual cycle and the other an annual cycle.

A recent work described how to distinguish between males and females of *A*. *antiqua* and reported the sex ratio to be 1:1 [25]. Therefore, it is considered relevant to update the information on the reproductive cycle of this species, since the last records were obtained between 25 to 40 years ago. In addition, we are currently in a context of global climate change, where determining factors of reproductive cycles may be changing.

Current knowledge about the parasitic fauna present in mollusc gonads, and their effect on population cycles, is limited [26]. A study in the clam *A*. *antiqua* indicated that 3.3% of the population was infected by cercariae of the family Plagiorchiidae [27]. Only gonads of females were infected, affecting the reproductive capacity of the species. This is the only work found on this species, indicating that information about parasitism is scarce. The present study was therefore designed to analyze the parasite load in the clam *A*. *antiqua*, to determine its temporal dynamic.

The general objective of the present work was to evaluate the reproductive characteristics of clam *Ameghinomya antiqua* in two natural beds located in areas of the southern Pacific coast, where the largest fisheries catches are made. The specific objectives were: (1) to describe variations in the gonadal cycle. (2) To report the Gonadosomatic and Condition Indices observed. (3) To analyse the parasite load in the clam *A*. *antiqua* with its molecular genetic identification at genus level. Finally, to determine the temporal dynamic of the parasite and evaluate how it could affect on the reproduction and population dynamics of this bivalve. This is important information for knowledge of this fishery resource, and the impacts of parasite infection on individuals, since it allows taking measures to protect or manage the resource based on its population dynamics.

## Materials and methods

### Samples

A total of 30 specimens of the clam *Ameghinomya antiqua* were collected every month from two locations on the Pacific coast in Chile: Carelmapu (41°49’S, 73°46’W) during the period September 2016 to July 2017, and Quellón (43°13’S, 73°33’W) during the period September 2016 to August 2017. The samples of November 2016 from Quellón were not sexed. Therefore, they could not be included in the analysis by sex. All sampled clams were of reproductive size (5.5 cms) or larger.

The samples were collected randomly by scuba diving. The individuals were dissected and subsequently soft tissue and gonads were separed. The total wet weight, soft tissue wet weight and gonad wet weight were recorded using a digital scale accurate to 0.01 g. A section of 1 cm^3^ was extracted from the mid-region of the gonad and fixed in 3% formalin solution for histological analysis.

### Gonadosomatic and Condition reproductive indices

The Gonadosomatic Index (GSI), i.e. the weight of the gonad as a proportion of the total body or flesh weight, was estimated by the following equation:

$${GSI}_{i}=100∙\frac{{GW}_{i}}{{BW}_{i}}$$

where *GW*_*i*_ is the gonad weight and *BW*_*i*_ is the body weight of individual “*i*”.

The well-being state of individuals was measured by the Relative Condition Index (RCI) proposed previously [28], which solves the problem of comparing individuals of different sizes and is suitable for intra-population comparisons [29].

$${RCI}_{i}=\frac{W_{i}}{W_{est}}$$

where *W*_*i*_ is the observed weight and *W*_*est*_ is the estimated weight based on shell size using the size-weight ratio of the population.

### Histological analysis

To obtain histological sections, the samples fixed in formalin were dehydrated using an ascending series of alcohols. They were then cleared in butanol and immersed in histological paraffin, using standard methods [30]. Finally, 7 μm sections were cut at 90° to the axis of the gonadal lobe. The sections were stained with hematoxylin-eosin and then observed in a Leica DM 750 microscope fitted with a Leica ICC50 W camera.

In order to characterise the gonad development stage of the sampled individuals, the histological sections were classified into one of three different states: “in development”, “ripe” and “spawned”, following the classification proposed for this species in previous studies [19].

### Parasitological analysis

The parasitological analysis was based on a sample of 200 individuals collected from the Carelmapu natural bed between October 2017 and January 2018. After the shell of the clam was removed, the animal parts (gills, digestive gland, mantle and gonads) were cut into several pieces using a pair of scissors and mixer with 0.85% saline. The mixture was washed several times with saline, and then each sample of tissue clam was examined to record the presence of parasites using a stereomicroscope. Some specimens were fixed in 70% ethanol and labelled individually for molecular studies.

The prevalence, mean intensity and mean abundance of infection in the samples analysed were estimated. The prevalence of infection is the number of hosts infected with one or more individuals of a determined species of parasite, divided by the number of hosts examined, expressed as a percentage [31]. The mean intensity of infection is the average number of individuals of a parasitic species found in a host, based solely on the individuals infected with a particular species of parasite [31]. Finally, the mean abundance of infection is the average number of individuals of a parasitic species found in a host, based solely on the total number of hosts examined, both infected and uninfected [31]. A Mann-Whitney U test was used to assess differences in the intensity and prevalence of infection between male and female hosts [32]. A contingency table with a Yates correction was used to evaluate differences in the prevalence between the sexes and months [32]. Correlations between maximum shell length and the intensity and prevalence of infection were evaluated with Spearman correlation tests [32]; for the analysis of prevalence, the host sizes were classified in 1-mm shell length classes.

### Molecular identification analysis

DNA was extracted from four parasites found in four different clams, using the Quick-DNA miniprep Plus kit. PCR amplification was carried out in three genes: 18S using the primers SB3 and A27 [33]; 28S with the primers C1 and D2 [34]; and ITS2 with the primers CC48 (5’TGTCGATGAAGAGTGCAGC’3) and CC49 (5’CAACTTTCCCTCACGGTACTTG’3) (Criscione Ch, personal communication). For each PCR, a final volume of 25uL was obtained. The PCR product was viewed in agarose gel at 1.5%. The final product was sent for sequencing in the Austral-omics (www.austral-omics.cl) core facility of Universidad Austral de Chile.

The sequences obtained were analysed and edited with the Geneious R10.2 software [35]. The consensus sequence was used for the similarity search in the database of the National Center for Biotechnology Information (NCBI, www.ncbi.nlm.nih.gov), using the BLAST software [36] to compare the sequences with those recorded in the database. All the sequences were aligned for phylogenetic analysis. The best evolution model for the complete data set for each gene was determined with the Mega v 6.0 software [37]. The Akaike information criterion (AIC) was used, showing that the model with the best fit was General Time Reversible + Gamma distribution (GTR + G). The RaxmlGUI v 1.5 software [38] was used for phylogenetic analysis, with Maximum Likelihood (ML) and Neighbour Joining (NJ) estimation. Finally, Figtree v 1.4.2 [39] was used to edit the phylogenetic tree. The external group selected was the closest available sister group [40]. Internal support for the nodes was assessed by 1000 bootstrap analyses.

## Results

### Gonadosomatic and condition reproductive indices

The Gonadosomatic Index (GSI) values showed the same trends between males and females in both locations. The two sites showed similar trends in mean GSI, however, a lag of one month was observed in the values between one locality and another.

**Fig 1:**
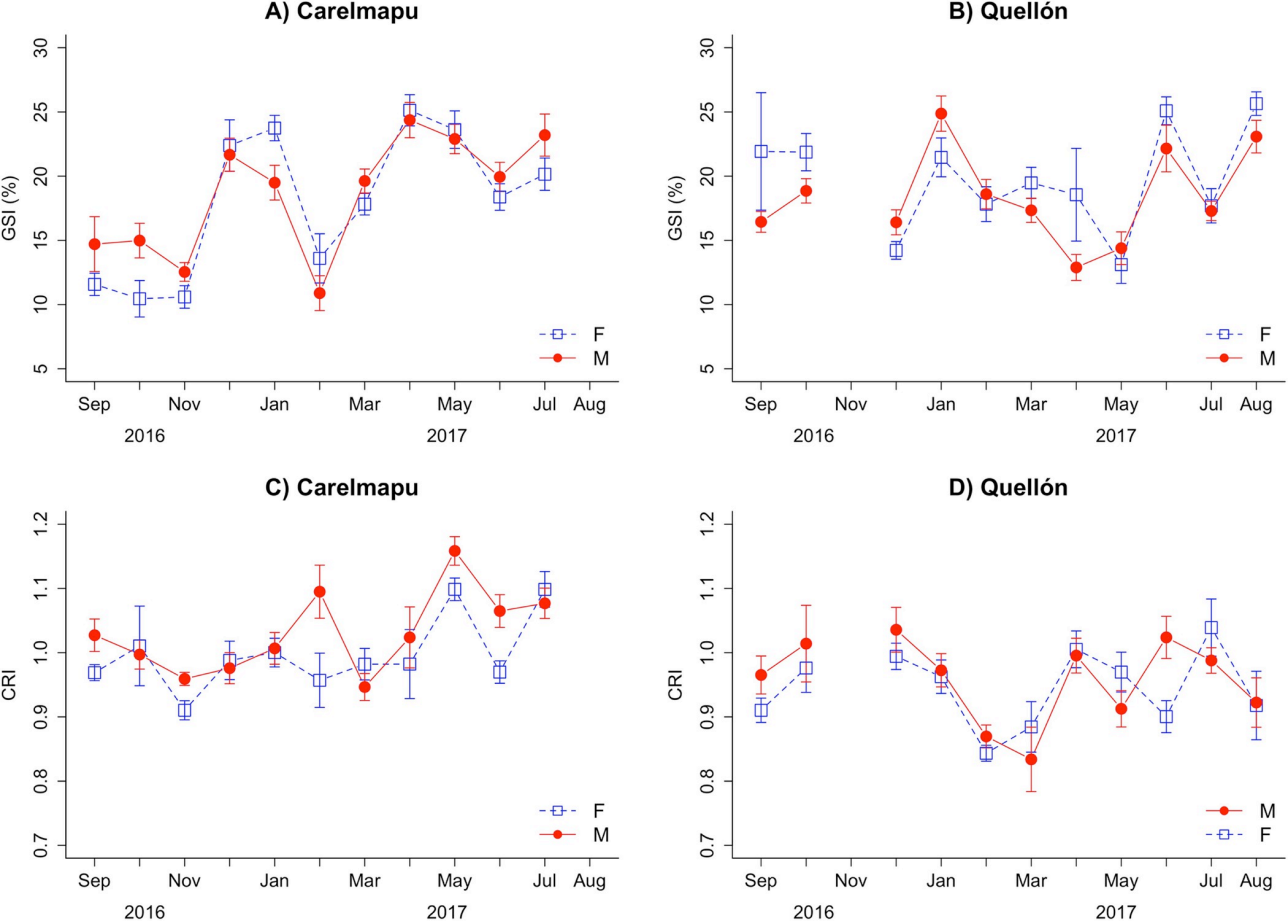
Monthly variation of the Gonadosomatic Index (GSI) and Relative Condition Index (RCI) of the chilean clam *A*. *antiqua* in natural beds. (A) Gonadosomatic Index in natural beds at Carelmapu. (B) Gonadosomatic Index in natural beds at Quellón. (C) Relative Condition Index in Carelmapu. (D) Relative Condition Index in Quellón. The error bars correspond to standard error.

At the Carelmapu site, synchronous reproductive behaviour were observed between male and female. This pattern began with the decrease of values in spring (September-November); an increase in the GSI was then observed in the early summer months (December-January), followed by a second, briefer decreased of values observed only in February (summer). The GSI then started to recover rapidly in March, reaching maximum values in the autumn and winter months (Fig 1A).

At the Quellón site, the GSI of males and females also presented synchronous behaviour with fluctuations. A quick decreased in the GSI value was observed in November, followed by recovery to high values in the summer months (December-January). Values decrease again in February and increase in the winter months (June) (Fig 1B).

The Relative Condition Index (RCI) for Carelmapu presented a decrease in spring (September-November), followed by a strong recovery in the early summer (December) and a second, briefer decrease in February (summer); this was more marked in females. A rapid increase started in April, reaching maximum values in the autumn and winter months (Fig 1C).

At Quellón, the RCI presented synchronic behaviour between the sexes. A rapid increase was observed in spring, more marked in females. A strong decrease in values occurred from January to March, followed by a recovery stage with fluctuations during the winter months (Fig 1D). No synchrony was observed between locations in the temporal RCI pattern.

The Pearson’s correlation between GSI and RCI was not significant, either at Carelmapu (Cor = 0.45; p-value = 0.141) or Quellón (Cor = 0.136; p-value = 0.658), despite showing similar increase and decrease trends during the analysis time.

### Gametogenic cycle

**Fig 2:**
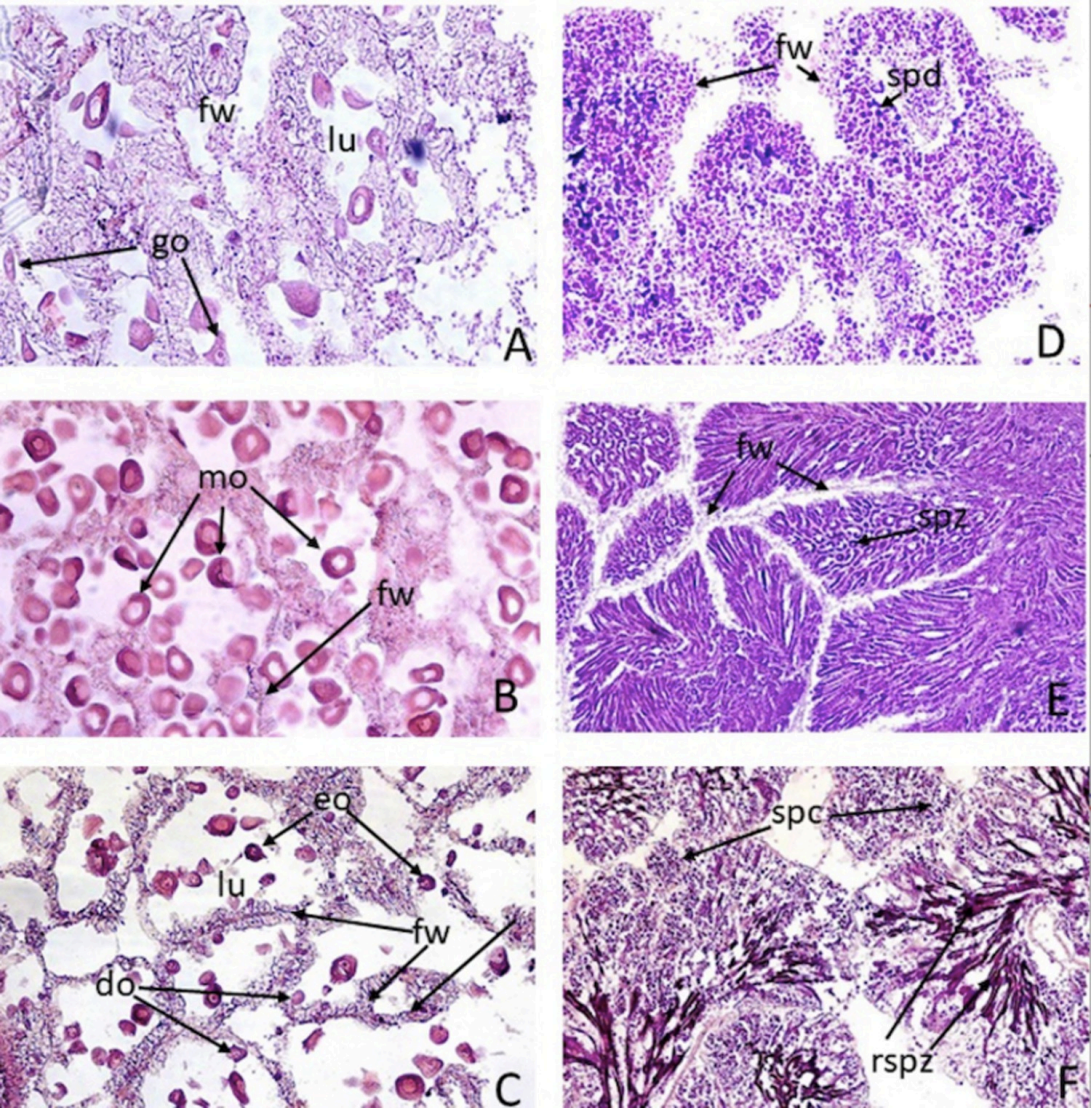
Microphotographs of gonadal states of *A*. *antiqua*. (A) "in development" female; (B) "ripe" female; (C) "spawned" female; (D) "in development" male; (E) "ripe" male; (F) "spawned" male. Microphotographs of cross sections of testicle and ovary stained with HE (1000X). Fw: follicular wall; spd: spermatids; Spz: spermatozoa; rspz: remains of spermatozoa; lu: lumen; eo: early oocytes; go: growing oocytes; mo: mature oocytes; do: deteriorating oocytes.

Three development states were observed in individuals sampled: “in development”, “ripe” and “spawned”, in both females (Fig 2A–2C respectively) and males (Fig 2D–2F respectively).

Females in the “in development” state presented slight growth of oocytes in the ovarian follicles, distributed inside a very small lumen. During this state, the oocytes have started vitellogenesis, as shown by the pale pink colour of some of the oocytes. The follicle walls and the surrounding connective tissue are well developed (Fig 2A). In the “ripe” state, the follicles appear highly developed, with abundant mature oocytes inside a narrow lumen. The oocytes have accumulated vitellus in their cytoplasm, which appears bright pink, while the nucleus is pale pink. As a result of the growth of oocytes inside the follicle, the follicular wall is less apparent (Fig 2B). In the “spawned” state, the females present empty follicles with a small number of oocytes in the follicular lumen; some of them have started cytolysis, while others remain in vitellogenesis. Much of the follicular wall is visible and highly developed (Fig 2C).

In males “in development”, the presence of follicles with a large number of spermatocytes and spermatids occupying most of the follicular walls is observed, while spermatogonia are barely visible (Fig 2D). In the “ripe” state the follicles appear highly developed, containing many mature spermatozoa with their heads against the wall of the follicle, and their tails towards the centre. The spermatozoa appear stained a pale colour. As a result of the advanced development of the follicles, the follicular walls appear small in size (Fig 2E). In the “spawned” state, remains of mature spermatozoa are observed in the lumen of the follicles, coloured bright violet. The follicle walls appear lined with maturing spermatocytes (Fig 2F).

### Reproductive cycle

**Fig 3:**
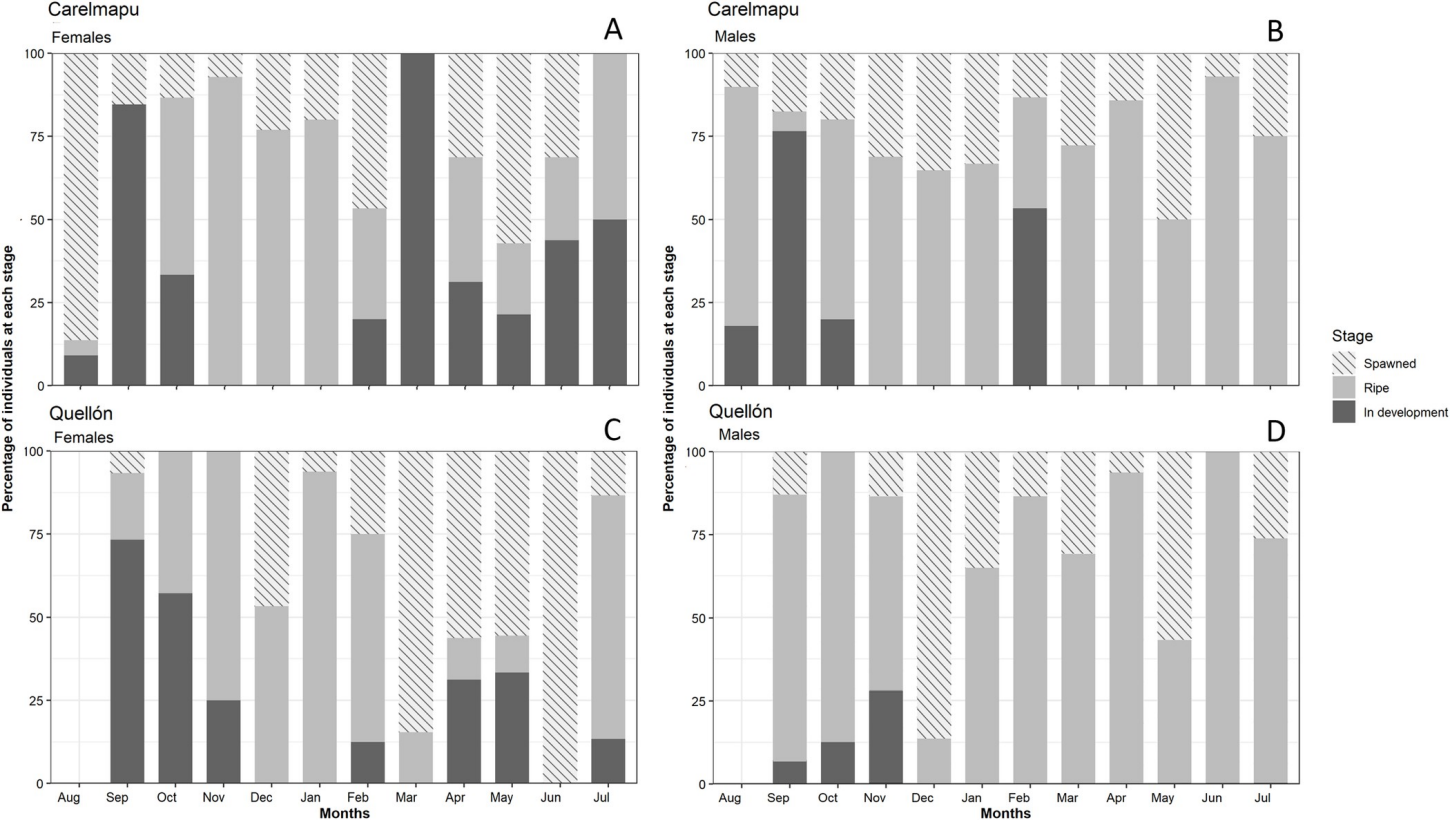
Temporal variation in the state of maturity in clams *A*. *antiqua*. (A) State of maturity of females at Carelmapu locality. (B) State of maturity of males at Carelmapu. (C) State of maturity of females at Quellón locality. (D) State of maturity of males at Quellón.

The reproductive cycle of the clam *A*. *antiqua* was recorder, separating by locality (Carelmapu and Quellon), by sex (female and male) and finally by gonadal stage (in development, ripe and spawned), which is observed in Fig 3 and the data is found in S1 Table. The gonadal cycle of specimens obtained from the population of *A*. *antiqua* at Carelmapu was remarkable because three significant increases of the “in development” state were recorded in females. The first occurred in September 2016, followed by a fast decrease to 0% in November; this first increase coincides with the period in which a significant increase was observed in males in the same state. The second increase started in February 2017 (summer) and reached the maximum frequency of individuals “in development” during March; the figure then fell during April to reach its minimum value in May. There was then a recovery of this state, with a third increase in July (Fig 3A).

The males presented two significant increases in the percentage of individuals “in development”; the first occurred September 2016 (early spring), followed by a drop to 0% in November to January. The second was observed in February 2017 (summer) (Fig 3B).

For the "ripe" state, an increase was observed in females from October 2016, high values were maintained throughout the spring and summer until February 2017 and a significant decrease in March was observed. A fast recovery of the gonadal state occurred during the following month (April) which was maintained until July, although with slightly lower values in the intermediate months (May and June).

Among males, the “ripe” state was high in August 2016, but fell sharply in the following month (September). Males in this state recovered to high values in spring and summer, from October 2016 to January 2017 (Fig 3B). In general, high percentages of "ripe" male individuals were observed throughout the year.

The number of “spawned” females was high in August 2016, decreasing in September and November. A series of fluctuations were then recorded in 2017 with increases in March, May and July followed by decreases in April and June (Fig 3A). In the Carelmapu population, the frequency of "spawned" males (Fig 3B) was low in August 2016, rose to a maximum in December 2016, and then fell in February 2017 (summer).

In the gonadal cycle recorded for Quellón natural bed of *A*. *antiqua*, a high proportion of “in development” females were recorded at the beginning of spring (September 2016), falling to 0% in summer (December and January). A slight recovery was observed in February 2017, but the value fell to 0% in March. "In development" females increased in April and May 2017, then fell back to 0% in winter (June). New "in development" females appeared in July 2017 (Fig 3C).

Among males (Fig 3D), the highest numbers of individuals "in development" was observed between September and November 2016. The proportion then fell to 0% in December and remained unchanged for the rest of the sampling period (until July 2017).

The observed frequency of "ripe" females was high in November 2016, January 2017, and July 2017, while the lowest percentages were observed between March 2017 and June (Fig 3C).

The number of “ripe” males was high in the September (spring) (Fig 3D) and in October then dropping in December. The proportion rose again in January, February, March, April and June (winter). High values were observed for "ripe" males at this location throughout the study period.

The proportion of “spawned” females at this site (Fig 3C) was low in September 2016 and during October and November. In the following months, the percentages of individuals in this reproductive state fluctuate showing a tendency to high values during the rest of the analysed time.

The frequency in males (Fig 3D) showed low values in in September 2016 and October. In November and December reaches the highest values of individuals in this reproductive state. The following months it fluctuates between lower values.

In histological analysis, was observed that some females sampled were infected with parasites. The histological sections of the infected gonads presented a similar histological structure to that of a normal gonad; however, at higher magnification of the microscope objective lens, a marked lack of gametes was observed in the gonadal follicles, and metacercariae parasites were observed in the follicle. The females with a high percentage of infestation in the gonads were discarded from their classification in the reproductive state, due to the difficulty of classifying them in any stage of development.

### Parasitological analysis

**Fig 4:**
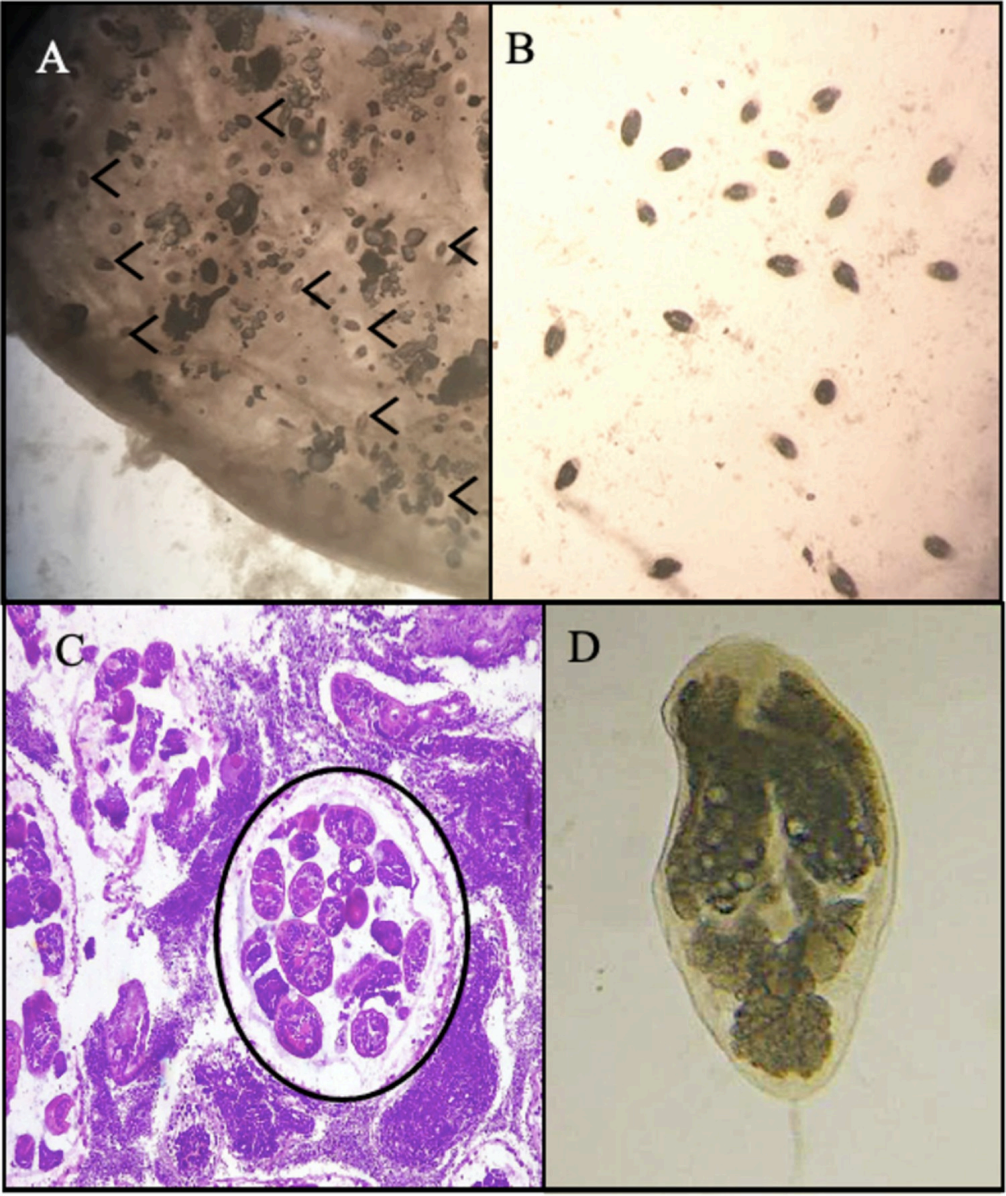
Parasites belong to the family Gymnophallidae collected from the clam *A*. *antiqua*. (A). Mantle tissue parasitized by the state of metacercariae observed with stereoscopic microscope at 1.5X. (B). Parasites recovered from the gonad tissue observed at 2.0X. (C). Photomicrograph of a gonadal section of a male with a high degree of parasitism, the indicated area shows segments of parasites located within one of the gonadal tubules. (D). Individual of Gymnophallid digenean observed with 40X (scale bar 40 μm).

Analysis of the tissues showed the presence of digenean parasites in the stage of metacercariae (Fig 4). The parasitological indices of prevalence, intensity and abundance are shown in Table 1. The mean number of parasites per clam was 33.8; the individual with the largest number had around 300 parasites. It was observed that 87% of the clams were infected in any of its tissues, with digenean parasites. The analysed indicators do not show differences between males and females. The Mann-Whitney U Test revealed no significant differences in the prevalence (p-value = 0.685) and intensity of infection (p-value = 0.886) by digenean parasites between male and female clams analysed. No significant differences were found in the prevalence (X-squared = 1.652, df = 3, p-value = 0.648) or intensity (X-squared = 1.652, df = 3, p-value = 0.648) of infection when analysed by sex and month, although a lower value of intensity and abundance of parasites is observed in the month of November. Finally, a positive correlation was found between prevalence and clam length (r_spearman_ = 0.452) and a negative correlation between intensity and shell length (r_spearman_ = -0.697).

**Table 1:** Parasitological indices.

	Prevalence (%)			Mean Intensity			Mean Abundance		
	Male	Female	Total	Male	Female	Total	Male	Female	Total
October	87.5	84.6	86%	41.4	50.4	46.0	36.2	42.7	39.6
November	88.3	88.5	86%	21.3	24.9	23.2	17.7	22.0	19.9
December	83.9	94.7	86%	55.3	43.3	51.5	46.4	41.0	44.3
January	96.0	84.0	90%	33.9	36.0	34.9	32.6	30.3	31.4
Total	88.9	87.9	87%	38.0	38.6	38.9	33.2	34.0	33.8

Parasitological indices of digenean parasites for October to December 2017 and January 2018 in the samples from Carelmapu.

### Molecular identification of parasites

**Fig 5:**
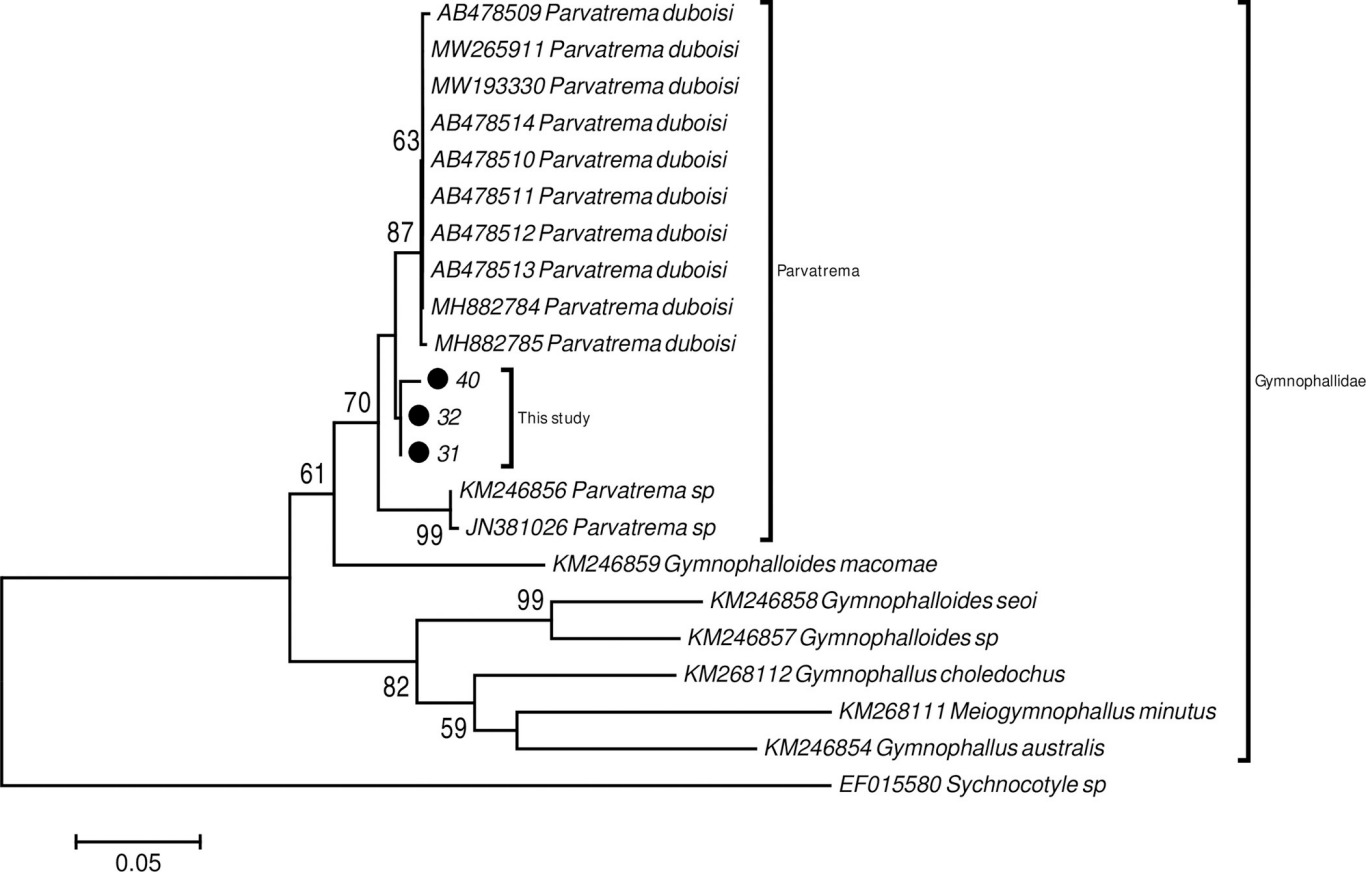
Phylogenetic reconstruction by NJ based on the ITS-2 region, for parasites collected from *A*. *antiqua*. The numbers along the branches indicate the percentages of the bootstrap support values resulting from the analyses of NJ. Codes indicate accession numbers for samples obtained from the GenBank.

Phylogenetic analysis was carried out separately for the three genes analysed (ITS-2; 18S rRNA and 28S rRNA). For analysis of the ITS-2 region, the samples were compared with GenBank sequences belonging to species of the genera *Gymnophalloides*, *Parvatrema* and *Gymnophallus*, using the genus *Sychnocotyle* as the outgroup (Fig 5). Analysis of 18S rRNA was based on sequences of digeneans of the genera *Gymnophalloides*, *Eurytrema*, *Pygidiopsis*, *Haplorchoides*, *Stellantchasmus*, *Tormopsolus*, *Brachycladium*, *Aurdistomum*, *Choanocotyle* and *Stephanostronum*; a species of the genus *Aspidogaster* was used as the outgroup (S1 Fig). Finally, for 28S rRNA, sequences of species of the genera *Gymnophallus*, *Parvatrema*, *Telorchis* and *Preptetos* were used, with a species of the genus *Aspidogaster* as the outgroup (S2 Fig). The topology recovered by the three genes was similar and grouped the parasites recovered in this study in a single clade together with species of the genus *Parvatrema* and close to *Gymnophalloides* (Fig 5); both these genera belong to the family Gymnophallidae of the superclass Digenea. The statistical ML bootstrap support for this clade was 97% for the 18S gene, 96% for the 28S gene and 86% for the ITS2 gene.

## Discussion

### Gonadosomatic and condition reproductive indices

The Gonadosomatic Indices (GSI) observed in Carelmapu and Quellón presented clear synchrony between sexes and similarity to the spawning and recovery pattern described previously for the species [19, 21, 22]. Although specific variations were found, probably associated with local environmental conditions, our results allow us to distinguish at least three rapid fall and recovery events in the GSI: the first, apparently longer, in spring (August-November), the second in summer (February, more marked at Carelmapu) and the third in winter (May-June). The variations in the intensity and precise moment of the fall and recovery of GSI between the two locations agree with the reports for this region of the authors cited above.

The Relative Condition Index shows a degree of correlation with the GSI which was not statistically significant. In general terms, however, it was observed that the physical condition of the clams improved in summer and autumn, coinciding with the greater food supply available as a function of the primary production cycles in southern Chile [17, 41, 42].

### Gametogenic and reproductive cycles

The results obtained show that *A*. *antiqua* presents a continuous gonadal cycle in both sampling locations, with at least three spawning periods during the present study, like a previous study in Ancud Bay [19]. At Carelmapu, spawning occurred at the end of winter (August), in summer (February) and in late autumn (May), when more than 50% of the population released mature gametes. At Quellón, spawning was recorded in early summer (December), early autumn (March) and winter (June). However, differing results have been observed in clams in other latitudes, with more clearly defined gametogenic cycles. For example, in the New Zealand clam *Mactra murchisoni* [43], synchronous gamete development between sexes was found, similar to this work, but with a marked winter spawning season (June in males and July in females).

It is well known that species that produce planktotrophic eggs require the availability of a sufficient supply of phytoplankton to enable the larvae to grow and metamorphose [44]. Thus, the biological significance of each spawning for *A*. *antiqua* reported here may differ, since not all spawning may be able to produce sufficient larvae for effective settlement. It may therefore be expected that only the larvae produced in the summer season (December-March for the Carelmapu population and February-April for the Quellón population) will be able to settle and form a cohort capable of achieving maturity. The reason for this is that the larvae of this clam settle approximately thirty days after spawning; moreover, the maximum growth rates occur between settlement and the beginning of March (end of summer), suggesting that the growth of the newly settled individuals of this species depends on both temperature and food supply [21]. The best growth rates occur during summer, and larval survival is probably also better during this period. From this it may be supposed that the spawning that occur in autumn and winter do not make a significant contribution to increasing the population of *A*. *antiqua*; however, this does not mean that no settlement occurs, but rather that the survival rate may be lower. This could be a strategy to help faster population recovery [19].

There is a no clear gonadal resting period; instead, there seems to be a constant process of gonadal recovery to produce gametes, as reported in other studies [21]. This rapid recovery of the gonads appears to be associated with the water temperature and the availability of particles of organic material (measured as concentration of chlorophyll-*a*) [21] due to the relation between gamete development and chlorophyll-*a* previously reported for clams [45, 46]. In the study areas of this work, however, the food supply fluctuates as both the organic and the inorganic seston follow a similar pattern to the temperature, i.e. high seston values are observed principally in the late spring [21]. The reports of phytoplanktonic blooms indicate seasonal variability in the phytoplanktonic biomass (chlorophyll–*a*) characterised by maximum ranges in the late spring and summer months, with a tendency to diminish in the autumn and winter [42]. These data, combined with those indicating that *A*. *antiqua* is a suspensivore species [41], feeding basically on material suspended in the water column, suggest that it may rely on other sources or strategies to obtain energy for its continuous gamete production. Sub-tidal populations live in environments with more stable temperatures and food supply [21], which would explain longer spawning periods. Another possible explanation of gonadal recovery and consequent spawning during periods with low food supply is that *A*. *antiqua* may have a flexible mechanism for obtaining food [47]; this would allow the species to act as a suspensivore when a good supply of suspended particulate matter is available, and as a depositivore when the supply is reduced [48]. Species which can act both as a suspensivore and a depositivore have been described among many taxa of invertebrates [49–51], including bivalves belonging to the superfamily Tellinacea [48, 52, 53].

The ratio between males and females in both the populations studied was close to the theoretical value 1:1, as described previously for this species [25] and for other species of bivalve molluscs. In both study sites, males spawned slightly before females; this suggests that the presence of spermatozoa in the medium may act as a stimulus to female spawning. The same pattern, with males spawning before females, has been recorded in New Zealand clams [43].

The reproduction cycle reported for this species is effectively continuous, however some differences are observed between the points of maximum recovery and maximum spawning found in the gonadosomatic and histological analyses. This may occur because the moments at which the maximum values are recorded may vary due to environmental factors.

### Parasitological analysis

In this work, infection of the clam *A*. *antiqua* by a digenean parasite of the family Gymnophallidae and supported by molecular data belong to the genus *Parvatrema* was identified. This family of parasites are being identified mainly parasitizing bivalves and their final hosts are coastal birds [54–56]. This parasite was found in great abundance in the clam population here examined. In general, the reported effect of the Gymnophallidae parasites in the host comprised the mantle and valve, and include hyperplasia and metaplasia of mantle tissue, inner shell surface alteration consisting in calcium carbonate deposits affecting the physiological performing [57, 58]. Indeed, it is interesting to note that the same prevalence and intensity were detected in both males and females in the months analysed. In some specimens the intensity of infection was so great as to cause gonadal castration, as reported in other molluscs in the Pacific Coast (*Mesodesma donacium*, [59]). Castration has a wide range of potential consequences on the host, including timing and success of reproduction, changes in distribution, and overall energy allocation dynamics [60], which can influence reproductive output [61, 62] at population level. In a recent study in the clam *Leukoma thaca* [63] a metacercaria was identified as belongs to the genus *Parvatrema*. The authors recovered metacercaria, sporocysts and cercaria stages of the same parasite, evidencing that this species uses *L*. *thaca* as the first and second intermediate host. In addition, the high prevalence of cercariae of *Parvatrema* sp in the gonads of this *L*. *thaca* suggests a potential detrimental effect at both the individual and population level. Probably, this genus of parasite is specific of clam and use then as unique intermediate parasite. The clam *A*. *antiqua* is an important resource for artisanal fishing along the coast of southern Chile, with both a socio-economic and a cultural significance. It is therefore important to understand this phenomenon to develop more effective programmes for sustainable exploitation which consider aspects that may affect the fertility of the species and might be important to the sustainability of natural bed of the species.

There is only one previous work that has reported metacercariae of digenean parasites, in the larval state, in samples of *A*. *antiqua* clams [26]; however, there are records of the presence of parasites in the gonads of other clam species [63, 64]. The paucity of knowledge about the parasites of marine bivalves of commercial interest hinders their identification at species level [59]. In particular, the immature states of few parasites have been studied in detail, and there is almost no information available on the life cycles of these endoparasites. The introduction, over the last decade, of molecular analysis techniques and the DNA barcoding method has paved the way to characterisation of the biodiversity of parasites, especially in bivalves of commercial interest where the occurrence of pathogens has been very little studied [54]. Thus, the results of the present study provide new knowledge of pathogens in natural environments which attack marine species of economic interest to artisanal fisherman on the Pacific coast.

The present work provides up-to-date information on the gametogenic cycle of this resource of great commercial importance, in locations where the greatest volumes are extracted, which becomes relevant in these times of global environmental changes. It identifies factors which favour population maintenance, such as continuous spawning throughout the year, as well as factors which may cause the population to decline, namely the detection of a parasite capable of castrating the gonads of these individuals. It is hoped that these results will provide basic information to support suitable measures for the management and protection of this resource. The management measures can be associated with the creation of restricted temporary periods (Veda) for the resource at times of greatest reproduction, or temporary closures associated with different peaks between locations. These results also allow defining strategies for repopulation the resource if it is necessary, identifying the appropriate periods for it, among other resource management measures that could be taken by the institutions of the State.

## Supporting information

S1 Table Gametic stage data by both localities (Carelmapu and Quellon) by sex (female and male) in the sampled time. (DOCX)

S1 Fig Phylogenetic reconstruction by NJ based on the 18S gene for parasites collected from *A*. *antiqua*. Codes indicate accession numbers for samples obtained from the GenBank. Bootstraps are indicated on the nodes.
(TIFF)

S2 Fig Phylogenetic reconstruction by ML based on the 28S gene for parasites collected from *A*. *antiqua*. Codes indicate accession numbers for samples obtained from the GenBank. Bootstraps are indicated on the nodes.
(TIFF)
