# Supplementary material for: Gonadal cycle, reproductive indices and detection of parasitism in the clam Ameghinomya antiqua in natural beds of importance for fisheries
Source: PLoS One. 2022 Apr 8;17(4):e0266538. doi: 10.1371/journal.pone.0266538 (PMC8992985; doi:10.1371/journal.pone.0266538)
Supplement: S1 Table — (DOCX) [file pone.0266538.s001.docx]

| Locality | Carelmapu | | | | | | Quellon | | | | | | | |
| --- | --- | --- | --- | --- | --- | --- | --- | --- | --- | --- | --- | --- | --- | --- |
| Gametic State | In Development (%) | | Ripe  (%) | | Spawned  (%) | | In Development  (%) | | | Ripe  (%) | | | Spawned (%) | |
| Sex | M | F | M | F | M | F | M | F | M | | F | M | | F |
| August | 17.9 | 9.1 | 71.8 | 4.5 | 10.3 | 86.4 | nd | nd | nd | | nd | nd | | nd |
| September | 76.5 | 84.6 | 5.9 | 0.0 | 17.6 | 15.4 | 6.7 | 73.3 | 80.0 | | 20.0 | 13.3 | | 6.7 |
| October | 20.0 | 33.3 | 60.0 | 53.3 | 20.0 | 13.3 | 12.5 | 57.1 | 87.5 | | 42.9 | 0.0 | | 0.0 |
| November | 0.0 | 0.0 | 68.8 | 92.9 | 31.3 | 7.1 | 28.6 | 25.0 | 57.1 | | 75.0 | 14.3 | | 0.0 |
| December | 0.0 | 0.0 | 64.7 | 76.9 | 35.3 | 23.1 | 0.0 | 0.0 | 13.3 | | 53.3 | 86.7 | | 46.7 |
| January | 0.0 | 0.0 | 66.7 | 80.0 | 33.3 | 20.0 | 0.0 | 0.0 | 64.3 | | 93.8 | 35.7 | | 6.3 |
| February | 53.3 | 20.0 | 33.3 | 33.3 | 13.3 | 46.7 | 0.0 | 12.5 | 85.7 | | 62.5 | 14.3 | | 25.0 |
| March | 0.0 | 100.0 | 72.2 | 0.0 | 27.8 | 0.0 | 0.0 | 0.0 | 68.8 | | 15.4 | 31.3 | | 84.6 |
| April | 0.0 | 31.3 | 85.7 | 37.5 | 14.3 | 31.3 | 0.0 | 31.3 | 92.9 | | 12.5 | 7.1 | | 56.3 |
| May | 0.0 | 21.4 | 50.0 | 21.4 | 50.0 | 57.1 | 0.0 | 33.3 | 42.9 | | 11.1 | 57.1 | | 55.6 |
| June | 0.0 | 43.8 | 92.9 | 25.0 | 7.1 | 31.3 | 0.0 | 0.0 | 100.0 | | 0.0 | 0.0 | | 100.0 |
| July | 0.0 | 50.0 | 75.0 | 50.0 | 25.0 | 0.0 | 0.0 | 13.3 | 73.3 | | 73.3 | 26.7 | | 13.3 |
