# Supplementary figures and images for: Gonadal cycle, reproductive indices and detection of parasitism in the clam Ameghinomya antiqua in natural beds of importance for fisheries
Source: PLoS One. 2022 Apr 8;17(4):e0266538. doi: 10.1371/journal.pone.0266538 (PMC8992985; doi:10.1371/journal.pone.0266538)

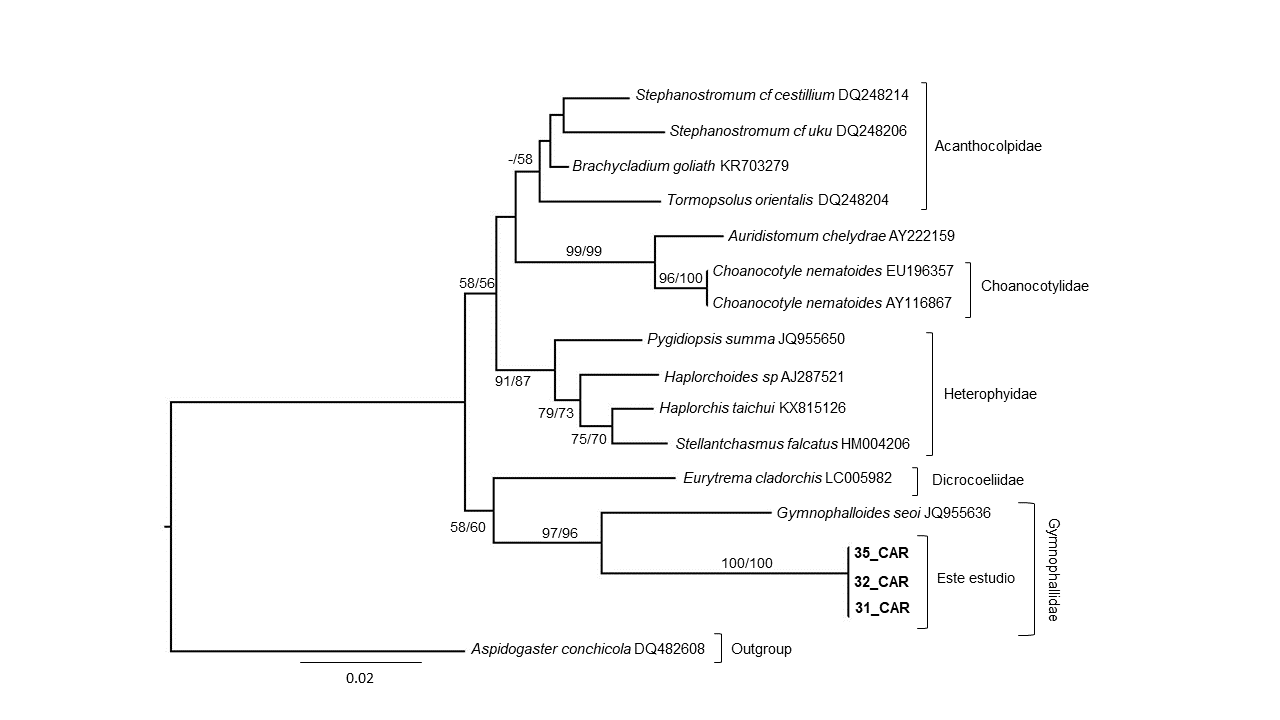

Supplement: S1 Fig — Codes indicate accession numbers for samples obtained from the GenBank. Bootstraps are indicated on the nodes. (TIFF) [file pone.0266538.s002.tiff]

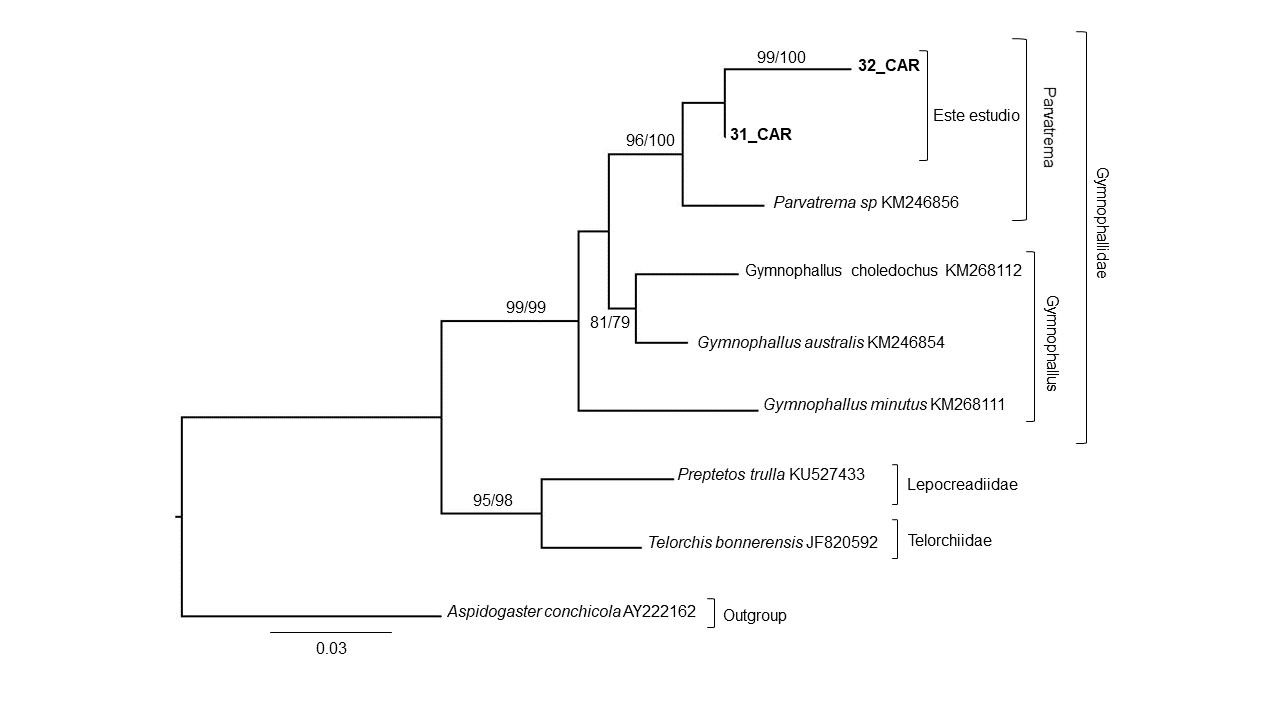

Supplement: S2 Fig — Codes indicate accession numbers for samples obtained from the GenBank. Bootstraps are indicated on the nodes. (TIFF) [file pone.0266538.s003.tiff]
